# Replacing Routine Oxytocin Infusion With Single-Dose Carbetocin at Elective Caesarean Section: A Service Improvement Evaluation of Postpartum Haemorrhage Outcomes

**DOI:** 10.7759/cureus.113782

**Published:** 2026-08-01

**Authors:** Savera Muslim, Sumaira Rafique, Suganya Sukumaran

**Affiliations:** 1 Obstetrics and Gynaecology, George Eliot Hospital NHS Trust, Nuneaton, GBR

**Keywords:** carbetocin, elective caesarean section, oxytocin administration, postpartum haemorrhage, quality improvement

## Abstract

Background

Routine postoperative oxytocin infusion after elective caesarean section can require prolonged intravenous administration, continued monitoring, and additional recovery-area resources. Single-dose carbetocin offers a simplified prophylactic uterotonic regimen, but local evaluation is important when implementing a change in practice.

Methods

This was a single-centre service improvement evaluation using anonymised aggregate data from women undergoing elective lower-segment caesarean section across two consecutive time periods. The oxytocin cohort included 392 elective caesarean sections performed between 1 August 2022 and 30 September 2023, during which routine prophylaxis consisted of oxytocin according to local practice, typically including postoperative infusion. The carbetocin cohort included 446 elective caesarean sections performed between 1 October 2023 and 31 December 2024, following implementation of single-dose intravenous carbetocin as routine prophylaxis. The primary clinical outcome was significant postpartum haemorrhage, defined as estimated blood loss greater than 1.5 L. The secondary clinical outcome was major postpartum haemorrhage, defined as estimated blood loss greater than 2 L. The service outcome was avoidance of routine postoperative uterotonic infusion.

Results

A total of 838 elective caesarean sections were included. Significant postpartum haemorrhage occurred in 10 of 392 cases in the oxytocin cohort and 12 of 446 cases in the carbetocin cohort, corresponding to rates of 2.55% and 2.69%, respectively. Major postpartum haemorrhage occurred in 2 of 392 cases in the oxytocin cohort and 4 of 446 cases in the carbetocin cohort, corresponding to rates of 0.51% and 0.90%, respectively. Following implementation of carbetocin, routine postoperative uterotonic infusion was no longer required as part of standard prophylaxis.

Conclusion

In this service improvement evaluation, replacing routine oxytocin infusion with single-dose carbetocin at elective caesarean section was associated with comparable rates of significant and major postpartum haemorrhage while simplifying postoperative uterotonic administration. The findings support the local safety of this practice change, although interpretation is limited by the retrospective aggregate design and absence of patient-level risk adjustment or additional morbidity outcomes.

## Introduction

Postpartum haemorrhage remains an important cause of maternal morbidity and mortality following childbirth and continues to require effective prevention strategies during caesarean section [1,2]. Uterine atony is one of the principal contributors to postpartum haemorrhage, and routine prophylactic uterotonic administration is therefore a key component of care after caesarean delivery [3,4].

Oxytocin has traditionally been used as first-line prophylaxis at caesarean section. In many units, this involves an intravenous bolus followed by a postoperative infusion, requiring ongoing intravenous access, infusion preparation, recovery-area monitoring, and nursing or midwifery time. Although this approach is well-established, prolonged postoperative infusion can add complexity to the elective caesarean recovery pathway, particularly in high-volume maternity services.

Carbetocin is a long-acting oxytocin analogue that provides sustained uterine contraction after a single intravenous dose. Its pharmacological profile allows uterotonic prophylaxis to be delivered without routine postoperative oxytocin infusion. Current National Institute for Health and Care Excellence (NICE) intrapartum care guidance recommends carbetocin by slow intravenous injection for women who have had a caesarean birth to prevent postpartum haemorrhage [5]. Previous systematic reviews and meta-analyses have shown that carbetocin is an effective alternative to oxytocin for prevention of postpartum haemorrhage after caesarean section, including elective caesarean delivery settings [6-9]. International consensus guidance also recognises oxytocin and carbetocin as uterotonic options during caesarean section [10].

Randomised and comparative studies have evaluated carbetocin against oxytocin for caesarean-section prophylaxis and have generally focused on additional uterotonic use, blood loss, uterine tone, and haemorrhage outcomes [11-13]. Economic modelling from a UK perspective has also suggested that carbetocin implementation may have pathway and resource implications, although local context remains important [14].

The present service improvement evaluation was undertaken after replacement of routine postoperative oxytocin infusion with single-dose intravenous carbetocin for women undergoing elective lower-segment caesarean section at a single maternity unit. The aim was to assess whether this practice change maintained comparable rates of clinically significant postpartum haemorrhage while simplifying postoperative uterotonic administration.

## Materials and methods

Design and setting

This was a single-centre service improvement evaluation using anonymised aggregate data from women undergoing elective lower-segment caesarean section. The project evaluated postpartum haemorrhage outcomes before and after a local change in routine uterotonic prophylaxis from postoperative oxytocin infusion to single-dose intravenous carbetocin.

The evaluation was conducted as a retrospective before-and-after comparison of two consecutive time periods. No patient-identifiable information was collected or reported, and no direct patient contact occurred. The manuscript was structured with reference to SQUIRE 2.0 (Standards for QUality Improvement Reporting Excellence) principles for reporting healthcare improvement work [15].

Eligibility criteria

The inclusion and exclusion criteria are presented in Table 1.

**Table 1:** Inclusion and exclusion criteria

Criterion type	Criterion
Inclusion	Elective lower-segment caesarean section performed during one of the predefined study periods
Inclusion	Procedures included in the anonymised aggregate service evaluation dataset
Exclusion	Emergency or non-elective caesarean section
Exclusion	Caesarean section performed outside the predefined study periods
Exclusion	Records not included in the anonymised aggregate service evaluation dataset

Study population and cohorts

Women undergoing elective lower-segment caesarean section during the defined study periods were included. Emergency caesarean sections were excluded. Women with known placenta accreta spectrum and cases with incomplete documentation of estimated blood loss were excluded.

The oxytocin/Syntocinon cohort included 392 elective caesarean sections performed between 1 August 2022 and 30 September 2023. During this period, routine prophylaxis consisted of oxytocin/Syntocinon according to local protocol, typically including a postoperative infusion lasting approximately 4 to 5 hours.

The carbetocin cohort included 446 elective caesarean sections performed between 1 October 2023 and 31 December 2024, following the change in local practice. During this period, routine prophylaxis consisted of single-dose intravenous carbetocin without routine postoperative uterotonic infusion, unless further treatment was clinically indicated.

Intervention

The service improvement intervention was the replacement of routine postoperative oxytocin/Syntocinon infusion with single-dose carbetocin for elective caesarean section. The intended pathway change was to maintain uterotonic prophylaxis while reducing the need for prolonged postoperative infusion, intravenous fluid administration, infusion equipment, and ongoing infusion-related monitoring.

This project did not evaluate an experimental medication strategy. It evaluated outcomes after implementation of an accepted prophylactic uterotonic regimen as routine care.

Outcome measures

The primary clinical outcome was significant postpartum haemorrhage, defined as estimated blood loss greater than 1.5 L. The secondary clinical outcome was major postpartum haemorrhage, defined as estimated blood loss greater than 2 L.

The service outcome was requirement for routine postoperative uterotonic infusion as part of the standard elective caesarean pathway. This outcome was evaluated at the pathway level because the change in practice removed routine postoperative oxytocin/Syntocinon infusion for women receiving carbetocin.

Statistical analysis

Data were analysed using anonymised aggregate outcome counts. Categorical outcomes were summarised as frequencies and percentages. For each postpartum haemorrhage outcome, the crude relative risk compared the carbetocin cohort with the oxytocin cohort as the reference group. Absolute risk differences were calculated as the difference in event rates between the carbetocin and oxytocin cohorts and are reported in percentage points.

Approximate 95% confidence intervals for relative risks were calculated using the logarithmic method for two-by-two contingency tables. Fisher’s exact test was used to compare event rates because the number of haemorrhage events was small. Statistical significance was interpreted using a two-sided p-value of less than 0.05. No patient-level adjustment, multivariable modelling, or subgroup analysis was possible because only anonymised aggregate service evaluation data were available.

The manuscript was structured with reference to SQUIRE 2.0 principles for reporting healthcare improvement work [15].

Ethical considerations

This project was conducted as a local service improvement evaluation using anonymised aggregate retrospective clinical outcome data. No patient-identifiable information was collected or reported, no direct patient contact occurred, and no intervention was performed for the purpose of research. Formal Research Ethics Committee approval and individual patient consent were not required according to local institutional governance arrangements.

## Results

Cohort overview and service outcome 

A total of 838 elective caesarean sections were included: 392 in the oxytocin/Syntocinon cohort and 446 in the carbetocin cohort. The cohorts were defined by consecutive time periods before and after the local change in routine prophylactic uterotonic practice. Before implementation, routine prophylaxis included postoperative oxytocin/Syntocinon infusion for approximately 4 to 5 hours. After implementation of carbetocin, routine postoperative uterotonic infusion was removed from the standard elective caesarean pathway. This represented a pathway simplification while observed rates of significant and major postpartum haemorrhage remained low.

The cohort description and service change are summarised in Table 2.

**Table 2:** Cohort description and service change

Variable	Oxytocin/Syntocinon cohort	Carbetocin cohort
Study period	1 August 2022 to 30 September 2023	1 October 2023 to 31 December 2024
Number of elective caesarean sections	392	446
Routine prophylactic uterotonic	Oxytocin/Syntocinon	Carbetocin
Route/regimen	Intravenous oxytocin according to local practice, typically including postoperative infusion	Single-dose intravenous carbetocin
Routine postoperative uterotonic infusion required	Yes	No
Main service change	Standard postoperative oxytocin infusion pathway	Simplified single-dose prophylaxis without routine postoperative infusion

Haemorrhage outcomes

Significant postpartum haemorrhage, defined as estimated blood loss greater than 1.5 L, occurred in 10 of 392 women in the oxytocin cohort and 12 of 446 women in the carbetocin cohort. This corresponded to rates of 2.55% and 2.69%, respectively. The crude relative risk was 1.05, with a 95% confidence interval of 0.46 to 2.41. The absolute risk difference was +0.14 percentage points, and Fisher’s exact test showed no statistically significant difference between groups.

Major postpartum haemorrhage, defined as estimated blood loss greater than 2 L, occurred in 2 of 392 women in the oxytocin cohort and 4 of 446 women in the carbetocin cohort. This corresponded to rates of 0.51% and 0.90%, respectively. The crude relative risk was 1.76, with a 95% confidence interval of 0.32 to 9.55. The absolute risk difference was +0.39 percentage points, and Fisher’s exact test showed no statistically significant difference between groups.

Postpartum haemorrhage outcomes are summarised in Table 3.

**Table 3:** Post-partum haemorrhage outcomes PPH = postpartum haemorrhage; CI = confidence interval Fisher's exact test was used because event numbers were small.

Outcome	Oxytocin/Syntocinon Cohort n=392	Carbetocin Cohort n=446	Relative Risk	95% Confidence Interval	Absolute Risk Difference	Fisher's Exact Test
Significant postpartum haemorrhage >1.5 L	10/392, 2.55%	12/446, 2.69%	1.05	0.46 to 2.41	+0.14 percentage points	p=1.00
Major postpartum haemorrhage >2 L	2/392, 0.51%	4/446, 0.90%	1.76	0.32 to 9.55	+0.39 percentage points	p=0.69

## Discussion

Principal findings

This service improvement evaluation found that replacing routine postoperative oxytocin infusion with single-dose intravenous carbetocin at elective caesarean section was not associated with an apparent increase in significant or major postpartum haemorrhage. Rates of estimated blood loss greater than 1.5 L were similar between the oxytocin and carbetocin cohorts. Major postpartum haemorrhage greater than 2 L remained uncommon in both groups.

The main service benefit of the practice change was removal of routine postoperative uterotonic infusion from the elective caesarean section pathway. This simplified prophylactic uterotonic administration and reduced the need for prolonged postoperative oxytocin infusion as routine care.

Interpretation of findings

The findings support the local safety of implementing single-dose carbetocin as routine prophylaxis for elective caesarean section. Although small numerical differences were observed, the absolute differences in postpartum haemorrhage rates were low, and confidence intervals were wide because event numbers were small. Therefore, the results should not be interpreted as proving equivalence between regimens. Rather, they suggest that the local change in practice did not produce an obvious increase in clinically significant haemorrhage when assessed using available aggregate outcome data.

The findings are consistent with the rationale for using carbetocin in elective caesarean section. Carbetocin provides sustained uterotonic activity after a single intravenous dose and avoids the need for routine postoperative oxytocin infusion. In a high-volume maternity setting, this may reduce complexity in the recovery pathway, although this project did not formally measure staff workload, recovery time, patient experience, or cost.

Service implications

For maternity units using routine postoperative oxytocin infusion after elective caesarean section, implementation of single-dose carbetocin may offer a practical service improvement. The intervention simplifies drug administration, removes the need for routine prolonged infusion, and may reduce demands on postoperative monitoring and infusion-related workflow.

In this evaluation, the pathway change was implemented without an apparent increase in significant or major postpartum haemorrhage. These findings may support other units considering similar pathway simplification, provided that local governance, staff training, and haemorrhage escalation protocols remain in place.

Strengths and limitations

A strength of this evaluation is that it reflects real-world implementation across consecutive service periods and includes a relatively large number of elective caesarean sections. The use of aggregate clinical outcome data allowed assessment of local safety after a unit-wide change in practice.

However, several limitations should be acknowledged. This was a single-centre retrospective service improvement evaluation and was not designed as a randomised or controlled study. Only anonymised aggregate data were available, meaning patient-level characteristics and haemorrhage risk factors could not be compared between cohorts. Baseline demographic and obstetric variables, such as maternal age, body mass index, parity, previous caesarean section, placental factors, birthweight, and preoperative haemoglobin, were not available. Blood transfusion, postoperative haemoglobin fall, additional uterotonic use, length of stay, recovery time, and cost outcomes were also not available.

Estimated blood loss is subject to measurement variation in routine clinical practice. In addition, because patient-level data were unavailable, adjusted analysis could not be performed. The findings should therefore be interpreted as a pragmatic local service evaluation rather than definitive comparative evidence of clinical equivalence.

Future work should include patient-level data, baseline haemorrhage risk factors, additional uterotonic use, transfusion, postoperative haemoglobin change, recovery pathway outcomes, and formal cost or resource evaluation.

## Conclusions

In this single-centre service improvement evaluation, replacement of routine postoperative oxytocin infusion with single-dose intravenous carbetocin at elective caesarean section was associated with comparable aggregate rates of significant and major postpartum haemorrhage. The practice change simplified postoperative uterotonic administration by removing the need for routine oxytocin infusion. These findings support the local safety and practicality of carbetocin implementation in the elective caesarean section pathway, although interpretation is limited by the retrospective aggregate design and absence of patient-level risk adjustment or additional morbidity outcomes.
